# Co-Constructing a Community-Based Telemedicine Program for People With Opioid Use Disorder During the COVID-19 Pandemic: Lessons Learned and Implications for Future Service Delivery

**DOI:** 10.2196/39236

**Published:** 2023-07-26

**Authors:** Stine Bordier Høj, Catherine de Montigny, Sofiane Chougar, Robert Léandre, Marie-Ève Beauchemin-Nadeau, Geneviève Boyer-Legault, Amélie Goyette, Sara-Kim Lamont, Julie Bruneau

**Affiliations:** 1 Centre de recherche du Centre hospitalier de l'Université de Montréal Montreal, QC Canada; 2 Service de médecine des toxicomanies Centre hospitalier de l'Université de Montréal Montreal, QC Canada; 3 CACTUS Montréal Montreal, QC Canada; 4 Department of family medicine and emergency medicine Université de Montréal Montreal, QC Canada

**Keywords:** opioid agonist treatment, opioid use disorder, medications for opioid use disorder, harm reduction, access to care, retention, telemedicine, telehealth, community-based services, opioid use, remote care, healthcare service, health care service, COVID-19, substance abuse, opioid disorder

## Abstract

The COVID-19 pandemic triggered unprecedented expansion of telemedicine, including in the delivery of opioid agonist treatment (OAT) for people with opioid use disorder (OUD). However, many people with OUD lack the technological resources necessary for remote care, have complex needs, and are underserved, with precarious access to mainstream services. To address the needs of these individuals, we devised a unique program to deliver OAT via telemedicine with the support of community outreach workers in Montreal (Quebec, Canada). The program was co-constructed by the service de médecine des toxicomanies of the Centre hospitalier de l’Université de Montréal (CHUM-SMT)—a hospital-based addiction medicine service—and CACTUS Montréal—a community-based harm reduction organization known and trusted by its clientele. All procedures were jointly developed to enable flexible and rapid appointment scheduling. CACTUS Montréal workers promoted the program, facilitated private on-site telemedicine connections to the CHUM-SMT, accompanied patients during web-based appointments if requested, and provided ongoing holistic support and follow-up. The CHUM-SMT offered individualized OAT regimens and other health services as needed. Overall, our experience as clinicians and community-based workers intimately involved in establishing and running this initiative suggests that participants found it to be convenient, nonjudgmental, and responsive to their needs, and that the implication of CACTUS Montréal was highly valued and integral to patient engagement and retention. Beyond the context of the COVID-19 pandemic, similar programs may present a flexible and accessible means to deliver alternative treatment options for people with OUD disengaged from traditional care, bridge gaps between communities and health providers, and improve access to care in rural or remote settings.

## Background

The field of telemedicine has a long-standing interest in improving the accessibility and quality of health care for remote and otherwise underresourced communities [1,2]. In response to the COVID-19 pandemic and associated physical distancing measures, the use of telemedicine modalities expanded exponentially to facilitate clinical services for a wide spectrum of health problems [3-5]. Continued integration into regular clinical care is likely, particularly in high-income countries [4,6,7], and could greatly improve service delivery for persons whose circumstances render in-person visits inconvenient or undesirable [7]. A lack of attention to the “digital divide” may, however, exclude groups that have been historically marginalized from experiencing these benefits and could even exacerbate health inequities [7-12]. Additionally, engaging such populations calls for approaches that reduce the social as well as physical barriers to accessing health providers [13-16]. This viewpoint paper describes how integrating a trusted community service into the framework of a hospital-based telemedicine service in Montreal (Quebec, Canada) created a program capable of engaging and retaining vulnerable people with opioid use disorder (OUD) in opioid agonist treatment (OAT) during the COVID-19 pandemic. We recount the success of this program as clinicians and community-based workers involved from its inception and by drawing upon patient testimonials, with a view to inform future service delivery for such populations.

## People With OUD, the COVID-19 Pandemic, and OAT Delivery via Telemedicine

People using illicit opioids experience a high burden of disease [17,18] and may be disproportionately impacted by major disruptive events such as a pandemic [19,20], particularly when facing other challenges such as housing instability [21,22]. Apart from risks related to COVID-19 exposure [23], public health responses to the pandemic directly impacted the risk environment shaping drug-related harms [24]: many low-threshold services that people with OUD rely on to meet essential needs or reduce harms associated with drug consumption, such as day shelters and supervised consumption sites, were forced to temporarily close or reduce their capacity, making already scarce resources even less accessible, while pandemic-related prevention measures also affected people’s ability to generate income, secure shelter, and obtain safe and secure supplies of drugs [25-29]. In Canada, these combined challenges were reflected in a 95% increase in opioid-related deaths in the first year of the pandemic (April 2020 to March 2021) compared with the year before [30].

Treatment with opioid agonists such as methadone or buprenorphine/naloxone is the first-line treatment for opioid use disorder [31,32] and can be highly effective in reducing illicit opioid use, preventing overdose, and improving multiple other health and social outcomes [18]. Nevertheless, inadequate treatment coverage and poor retention persist worldwide [18,33], with evidence for suboptimal and restrictive delivery practices (eg, inadequate medication dosing, poor access to unsupervised or “take-home” doses, and frequent urine drug screening) in many settings [34,35]. In Canada, as elsewhere, OAT is highly regulated [36] and typically delivered within health institution settings [37]. Prior to the pandemic, an estimated 66% of people who inject drugs nationwide, and 44% of those in Quebec, were receiving OAT [38]. People with OUD in Montreal had called for greater service outreach in places in which they spend their time, while emphasizing increased patient autonomy, flexible scheduling, diversified treatment options, and the implication of peer workers as ways to improve OAT services [39]. Indeed, participants in our program often recounted unsatisfactory experiences with conventional OAT services, describing a lack of responsiveness to their needs and experiences, ineffective communication, an absence of participatory decision-making, and stigmatization, alongside difficulties with medications and dosing, withdrawal symptoms and side effects, and rigid service rules.

OAT delivery in Canada is bound by federal regulations under the Controlled Drugs and Substances Act (CDSA), while provincial and territorial governments regulate the scope of practice for health professionals [40]. Following the declaration of the COVID-19 pandemic in March 2020, federal health authorities issued emergency legal exemptions to the CDSA, enabling verbal prescription of controlled substances alongside expanded authorizations for pharmacists to extend, renew, and transfer such prescriptions [41]. Meanwhile, to support the implementation of best practices, federal health authorities commissioned the Canadian Research Initiative in Substance Misuse (CRISM) to develop a series of national guidance documents, including recommendations for the use of telemedicine in addiction services [42] and prescribing of psychoactive substances to support people with OUD who need to self-isolate [43]. The medical regulatory authority of Quebec rapidly updated their OUD treatment and telemedicine guidelines to integrate these changes, relax restrictions on unsupervised dosing of long-acting opioid agonists, and endorse the use of telemedicine for OAT initiation and continuation [44-46]. Quebec-specific expert recommendations for OAT delivery, including guidance for “safer supply” prescriptions of short-acting opioids, were subsequently released in October 2020 [47]. Together, these changes represent a seismic shift in the regulatory structures and guidance shaping treatment for people with OUD [48]. Evidence suggests a broad uptake of telemedicine prescribing in Canada, with over half of OAT clinics surveyed offering this service by June 2020 [49].

## Recognizing a Need for Further Adaptation

The Centre hospitalier de l’Université de Montréal (CHUM) is an academic hospital in downtown Montreal, whose addiction medicine service (Service de médecine des toxicomanies [CHUM-SMT]) offers an integrated model of care for people with substance use disorders and complex social and medical needs. This model spans a wide range of interventions, from harm reduction to specialized multidisciplinary medical and psychiatric treatment, and includes a low-barrier OAT program. From March 2020, OAT practices were rapidly amended in line with the aforementioned recommendations to shift outpatient consultations to web-based or telephone visits and allow initiation of new patients without requiring that they visit the outpatient clinic. Like clinicians in other settings [50-52], however, we noted that access to OAT remained inequitable under a telemedicine model, particularly for individuals unable to access a phone or computer or are otherwise unwilling to access hospital-based services for fear of stigmatization [8,53].

Recognizing the need to actively reach out and build trust with this population that has been socially marginalized, we created a new partnership with a community-based harm reduction organization, CACTUS Montréal. Located in the core of downtown Montreal, CACTUS Montréal is home to one of North America’s oldest needle-syringe programs and takes a pragmatic and humanistic approach to serving people who use drugs through prevention, education, and leisure activities. It was also one of the few such services to remain open in Montreal during the initial months of the COVID-19 pandemic. Together, we co-designed a novel model of care premised on offering easily accessible, high-quality telecare for people with OUD with the support of the CACTUS Montréal community workers they trust.

Procedures were developed jointly to enable a flexible and rapid appointment scheduling system that would meet the needs of this population while ensuring confidential flows of patient information. CACTUS Montréal community workers informed their clients of the program, facilitated initial eligibility screening, and helped interested clients book an on-site web-based appointment with the CHUM-SMT team, generally within the same week. Participants thus remained physically within the CACTUS Montréal facilities, where they could not only access the technological resources and assistance necessary to engage in care but also draw on the support of community workers during and after their appointment. This enabled warm hand off from a trusted person to the CHUM-SMT and equally created an environment in which patients could easily pose questions, reschedule appointments, and follow up with staff about the program throughout their participation. Clinics ran on Tuesday and Wednesday afternoons, allowing some walk-in appointments as needed.

During the web-based appointments, the CHUM-SMT team (comprising a physician and a nurse) evaluated each patient and initiated an individualized treatment regimen. To foster engagement in care, treatment was tailored to the participants’ particular needs and their prior experiences with OAT. An initial regimen typically included a long-acting opioid (eg, methadone, buprenorphine/naloxone, or extended-release morphine) combined with a short-acting opioid (eg, hydromorphone) to manage withdrawal, increase comfort, and reduce the risk of illicit opioid use and overdose during initiation. Prescriptions were sent by fax directly to the participant’s local pharmacy and a follow-up appointment was immediately scheduled. Importantly, the CHUM-SMT team could also assess and treat patients’ other medical needs, with CACTUS Montréal nurses available to draw blood samples (eg, for HIV or hepatitis C testing) as necessary. Participant consent was obtained from the outset to define the nature of information exchange between CHUM-SMT and CACTUS Montréal, who communicated frequently to schedule appointments based on the participants’ needs.

To support implementation, both partners entered a formal collaboration agreement and devised a tool kit including a shared appointment calendar, patient evaluation and referral forms, user guides for connecting to various telemedicine tools, and trainings in the web-based clinic and on OAT for CACTUS Montréal staff. Patient flows were managed with assistance from the CHUM Network Flow Optimization Center (Centre d’optimisation des flux reseau)*,* a service promoting accessibility and continuity of care through various measures including coordination of linkages to the broader health network and telehealth services.

## Successes, Challenges, and the Importance of Building Institutional Relationships

A major challenge at the time of implementation was to rapidly organize the aforementioned trainings, tools, and procedures so that CACTUS workers could appropriately support clients initiating OAT through the service. This effort was facilitated by a previously well-established working relationship and institutional support from both partners, with our first patient enrolled just 30 days after the first planning meeting and 45 days after declaration of the COVID-19 public health emergency in Quebec.

The initiative was viewed as transformative by the staff and enthusiastically received by the participants, who, we believe, found the program convenient, flexible, and responsive to their needs despite some reservations about the “impersonal” nature of telemedicine. In our view, the implication of CACTUS Montréal and its community workers was critical to creating an environment of trust, confidence, and nonjudgment; maintaining open lines of communication between appointments; and enabling access to care within a setting that participants were already frequenting. Many participants had long-standing ties and positive relationships with CACTUS Montréal, which contrasted starkly with testimonies of their unsatisfactory experiences with mainstream health services. This appeared to facilitate engagement and lend legitimacy to the program, with telemedicine being a tool enabling us to quickly and easily reach beyond the confines of the conventional health system. Unlike programs delivering OAT through telemedicine alone, our hybrid model also enabled easy access to laboratory testing and subsequent initiation of care for other chronic health problems (eg, HIV and hepatitis C virus) affecting this population.

Partnerships between institutional health services and independent community organizations can be complex to develop because the community approach to health, particularly in the area of harm reduction and prevention of sexually transmitted and blood-borne infections, has developed in response to the inability of traditional services to reach and maintain ties with groups that have been historically marginalized. In this case, a history of constructive engagement between the CHUM and CACTUS Montréal—also in the context of various prior research initiatives—was the critical foundation that allowed us to respond quickly and positively to the disruption caused by the COVID-19 pandemic. Our telemedicine initiative has further strengthened these ties, marking a significant step toward further innovations in service delivery. Indeed, the program proved so popular, it has now expanded to a second harm reduction organization in Montreal. Mobilizing the technical and human resources needed to establish new partnerships and meet client demand has been taxing and confirms a need to develop longer-term plans and infrastructure so that the model, once fully evaluated, can be deployed more broadly.

## Conclusion

Facilitated by the COVID-19 pandemic and the ensuing amendments to federal and provincial regulations and recommendations for best practices, our community-based telemedicine program succeeded in bringing OAT to people with OUD within a space where they feel comfortable and accepted. The implication of CACTUS Montréal, an ally who accompanies clients through difficulties at their own pace and based on their own needs and priorities, was fundamental to engaging our patients and responded to priorities previously expressed by local people with OUD.

It is imperative that such initiatives and the emergency-led legal and structural contexts underpinning them persist, evolve, and expand well beyond the pandemic. Emerging evidence suggests that both patients and providers support the continuation of regulatory reforms and telemedicine delivery of OAT and perceive these as facilitating individualized patient-centered care [54,55]. More programs enabled by these innovations must be quickly and thoroughly evaluated to build an evidence base for their safety and effectiveness and guide further adaptation. Investments must then allow the science to move alongside implementation, scale-up, and formalization of such novel care pathways toward a more agile and adaptive response to the continually evolving overdose crisis [56,57]. The success of vaccine development and scale-up initiatives amid the pressing threat of the COVID-19 pandemic, as well as the rapid deployment of telemedicine and other sweeping societal interventions, has demonstrated our capacity to innovate and implement simultaneously. Reducing overdoses among people with OUD deserves the same sense of emergency and commitment to innovative thinking. We firmly believe that programs such as ours can reduce health inequalities and save lives by meeting people “where they are,” bridging gaps to institutional services and shifting patient-provider relationships toward a place of mutual understanding and holistic engagement [58].

## Abbreviations

CDSA: Controlled Drugs and Substances Act
CHUM: Centre hospitalier de l’Université de Montréal
CHUM-SMT: service de médecine des toxicomanies of the Centre hospitalier de l’Université de Montréal
CRISM: Canadian Research Initiative in Substance Misuse
OAT: opioid agonist treatment
OUD: opioid use disorder
